# What ails tuberculosis notification from the private sector in urban India: a qualitative study among stakeholders in Bengaluru City, southern India

**DOI:** 10.3389/fpubh.2025.1531069

**Published:** 2025-04-04

**Authors:** Anand D. Meundi, Jan Hendrik Richardus

**Affiliations:** ^1^Department of Community Medicine, Dr. Chandramma Dayananda Sagar Institute of Medical Education and Research (CDSIMER), Dayananda Sagar University (DSU), Bengaluru, India; ^2^Department of Public Health, Erasmus MC, University Medical Center, Rotterdam, Netherlands

**Keywords:** tuberculosis notification by specialists, private providers, medical colleges, India, experiences

## Abstract

**Background:**

Tuberculosis (TB) case reporting is vital for national and subnational surveillance and is mandated in India since 2012. All health providers, public and private, must report through the web-based system, “Nikshay.” The prevalence: notification ratio from 2019 to 2021 was 2.84 (prevalence was almost 3 times the notification), suggesting under-notification. This study explores the experiences, identifies barriers and facilitators, and examines perceptions regarding mandatory notification and incentives among private providers (PPs) in diverse contexts in Bengaluru city.

**Methods:**

Focused group discussions were held with specialist faculty of Pediatrics, Internal Medicine, and Pulmonology across three medical colleges and in-depth interviews included freelancing specialists and general practitioners. Data was collected in Bengaluru from January 2018 to July 2019, analyzed using a framework approach, referencing social learning theory, precede/proceed model, and theory of learned behavior. Thematic content analysis linked emerging themes to codes.

**Results:**

There was unmet expectation regarding lack of feedback from the NTEP regarding the patients notified. Emphasis on bacteriological diagnosis for every patient by NTEP deterred notification. Incentives were felt either to be insufficient for doctors or the PPs felt they were obliged to the national programme to notify. Barriers included obligation to maintain confidentiality, stigma, lack of knowledge of how to notify and facilitators included being recognized for their efforts and implementation of compulsory notification in letter and spirit.

**Conclusion:**

Strategies to minimize stigma through education of patients at diagnosis and regular communication with private providers about the notification process, guidelines, and policy improvements can decrease resistance to notification. Recognizing best practices and rewarding on professional platforms could motivate private providers, alongside continued monetary incentives. Finally, demonstrating effective implementation of mandatory notification provision may boost private providers’ morale.

## Introduction

According to World Health Organization’s Global Tuberculosis (TB) Report 2023, India accounted for the highest number of TB cases in the world in 2022, making up for about 27% of the global burden (1). TB case reporting is the backbone of tuberculosis surveillance at the national and subnational levels (2). TB was declared a notifiable disease by the Government of India in 2012 (3). This requires all public and private health providers in the country to notify all TB patients who they diagnose/treat. A recent TB prevalence survey in India showed that approximately 50% of patients with TB symptoms consult private health care facilities (4). TB Notification by the government and private providers in India is performed under the National TB Elimination Programme (NTEP) through a web-based case-based reporting system with a dedicated online website called “Nikshay.” A country-wide study of TB notification rates in India in 2017 estimated that only 2.96% of parliamentary constituencies in India had met the 50% target of private sector notification (5). As of March 2022, approximately 51,000 private health facilities were registered under Nikshay. In 2021, approximately 2.1 million TB patients were diagnosed in India; 1.4 million (68%) were from the government sector, and 0.6 million (32%) were from the private sector. During the same period, 13,559 patients were reported in Bengaluru city (Bruhat Bengaluru Mahanagara Palike area), 7,909 (58%) of whom were from the government sector and 5,650 (42%) of whom were from the private sector (6). Between 2019 and 2021, the prevalence: notification ratio was 2.84, suggesting that for every TB case detected and reported, 2.84 cases are either not diagnosed or nor reported (4). Strengthening the TB notification system and ensuring that all cases are reported can help in better understanding the true burden of the disease and in planning appropriate interventions.

There is growing evidence that TB notification from private providers (PPs) is determined by several factors, the most important being the experience of PPs during the process of notification. These experiences have been found to be related to ease of the process, familiarity with technology, internet availability, glitches in the Nikshay portal, time taken to complete the process, and the availability of intermediaries who can troubleshoot during difficulties (7, 8). Lack of trust in the NTEP and the absence of positive feedback from the NTEP in the form of acknowledgment have also been found to deter notification (9). Therefore, the present study aimed to (1) document the experiences of PPs during the process of notification, (2) elicit barriers, facilitators and suggestions concerning TB notification from PPs, and (3) explore the perceptions of PPs regarding mandated compulsory notification and incentives for TB notification. PPs in India who treat TB work in various settings—specialists in medical colleges, freelancing specialists, hospital administrators, and general practitioners. The milieu for notification of TB patients is not the same in these settings. Therefore, in the present study, PPs in these varied settings in Bengaluru city were included.

## Materials and methods

Focus group discussions and in-depth interviews were conducted with the PPs to obtain their views on the notification of TB patients. The PPs belonged to three categories: specialist faculty of medical colleges, freelancing specialists, and general practitioners. All PPs were employed/practiced in Bengaluru city under the city corporation, Bruhat Bengaluru Mahanagara Palike (BBMP) limits. Eight focus group discussions (FGDs) were conducted with the faculty of medical colleges specializing in pediatrics, internal medicine, and pulmonology. Three medical colleges were included. Each FGD group consisted of a single specialty and participants belonging to all academic cadres, namely, senior residents, assistant professors/associate professors and professors. Twenty-four in-depth interviews (IDIs) were conducted with free lancing specialists, which included 6 pediatricians, 6 internal medicine specialists and 6 pulmonologists (all outside medical colleges); 4 IDIs with general practitioners (with only an MBBS degree); and 2 IDIs with hospital administrators (Table 1). The participants for the FGDs were purposively selected from the three medical colleges for each of the specialties. For the IDIs, the first participant in the particular specialty was purposively selected and subsequent participants in that specialty were selected using snowball sampling. None of the potential participants who were contacted refused to participate.

**Table 1:** Demographic details of the participants.

Sl. No.	Qualitative method	Participant characteristics	Total participants
1.	Focus Group Discussion (specialist faculty working in Medical Colleges)	Professors/Associate Professors Males: 15 Females: 8 Assistant Professors/ Senior Residents Males: 20 Females: 9	52
2.	In-Depth Interviews (freelancing specialists/general practitioners/hospital administrators)	Internal Medicine specialists Males: 6 Females: None Pulmonology specialists Males: 4 Females: 2 Pediatric specialists Males: 6 Females: None General practitioners Males: 5 Females:1 Hospital administrators Males: 2 Females: None	26

The FGDs and IDIs were conducted between January 2018 and July 2019 by the first author (male, faculty of Community Medicine in a private medical school in a neighboring state qualified with MBBS, MD) using an interview guide (Table 2) until a saturation point was reached, at which point no new information was forthcoming. Reflexivity was applied during the data collection and analysis. The interview guide was pilot tested before actual use. The first author has been trained in qualitative methods and has published qualitative research previously. Written informed consent was obtained prior to the interview. There was a formal introduction before the FGDs/IDIs started, and the participants were informed about the objectives of the present study. The FGDs lasted for an average of 50 min, and each IDI lasted for approximately 25 min; the interviews were conducted in English, were audio recorded, and were transcribed later. All FGDs were facilitated by the first author. Note-taking during FGDs was done by the same person who also transcribed the audio recordings. The FGDs/IDIs were held at the participants’ workplace. The interviewer ensured that the interaction took place with prior appointment at an isolated area, away from interference.

**Table 2:** Topic guide for conducting FGD/IDI.

Topic: Perceptions about TB notification under Ni-Kshay
Main questions What has been your experience with notification of cases of TB? What do you think are the factors that are preventing and enabling private doctors from notifying TB? What do you think are the changes in the TB notification procedures that can increase notification of TB by doctors in the private sector? What do you think will be the effect of compulsory TB notification mandate on notification volume and quality? What do you think will be the effect of incentives for TB notification on notification volume and quality? Follow-up questions Probes

A framework approach was used for the data analysis (10). The themes for framework analysis were derived from the constructs (listed in brackets) of three theories: (1) the social learning theory (behavioral capability, expectations, self-efficacy, observational learning, reinforcement and social support), (2) the precede/proceed model (positive predisposing factors, enabling factors and reinforcing factors), and (3) the theory of planned behavior (attitude towards the behavior, subjective norms and perceived behavioral control) (11, 12). These constructs were listed as initial codes for data analysis and additional themes were identified as the analysis progressed. Thematic content analysis was performed to link the emerging themes with the codes. Triangulation was used in the analysis by cross-verifying themes across the FGDs and IDIs.

Two coders performed the content analysis. A licensed version of NVivo Pro Version 12 was used for qualitative data analysis and management. The COREQ criteria for reporting qualitative studies were adopted for presenting the qualitative results of the present study (13). The credibility of the data was ensured by respondent validation. Two specialists who participated in the FGDs were selected randomly. These selected participants were given a draft of the report of this study for their critical comments. The comments obtained were integrated into the analysis and interpretation. The audit trail was maintained to ensure confirmability. Written informed consent was obtained from participants of all the above studies prior to data collection. Ethical approval was obtained from the institutional review board of Pariyaram Medical College, Pariyaram (now renamed Government Medical College, Kannur), Kerala, India (letter reference no. G1.2747/12/ACME), where the author was working at the time of data collection.

## Results

The results are presented under the broad themes and sub-themes. The organization of the themes and subthemes that emerged from the thematic content analysis are presented in Table 3.

**Table 3:** Structure of themes and subthemes with references to the theories considered in the framework analysis.

Expectations from NTEP (social learning theory)	Self-efficacy in notification (social learning theory, theory of planned behavior)	Reinforcement (social learning theory)	Enabling factors (precede/proceed model)	Barriers (precede/proceed model)	Suggestions
Data from notifications must be analyzed and used. No feedback after notification.	Bacteriological diagnosis is difficult in pediatric patients.	Differing opinions on incentives reinforcement: The incentive option might be misused. Not necessary. It is their duty. The incentive is not enough. Incentives do not make a difference for most doctors, may be attractive for health workers and pharmacists. Recognition of doctors as notifiers more important than incentive. Club incentives with enforcement. Incentive for notification not being disbursed.	Diagnosis simpler using NTEP guidelines in pediatric cases.	Confidentiality and stigma. No NTEP hub in corporate hospitals. Lack of knowledge of notification process.	List laboratories where testing for TB is being done and actively look for notification from them. Pharmacies must maintain a line list of patients to whom anti TB medication is dispensed. Get notifications through professional associations.

### Expectations from the NTEP

The PPs strongly felt that no “action” was being taken on the data that were obtained from notification of TB. They expected the NTEP to utilize notification data to trace contacts, screen them for TB and contribute to TB control. There was no feedback from the NTEP regarding the patients notified, which further frustrated the PPs.

#### NTEP is not using notification data for TB control

*I am okay for anything to notify. The only thing is they should do something with that data. Just notifying and collecting the data does not do anything. Somebody has to go and do case contacts, somebody has to screen, expose people, somebody has to go and find the index cases, somebody has to do all that, that is the government’s part. If they are not doing that, the problem of TB will never be solved. That is not my job but until now, it is not done* (Free lancing pulmonologist).

#### No feedback from NTEP about cases notified by me

*I am not writing anything. I am just referring to the NTEP with a letter and after that, I have never received anything in return whether the patient has gone there or not. I have no trace of any of the patients whomsoever I referred* (Freelancing pediatrician).

### Self-efficacy in diagnosing TB

A common experience of the pediatricians was that bacteriological diagnosis of TB, which was rigidly sought by the NTEP, was difficult. This resulted in many of the patients being notified and referred from PPs being subjected to laboratory investigations again in NTEP laboratories. However, the pediatricians acknowledged that in recent times, the NTEP had started accepting clinical diagnosis for patients starting anti-TB treatment.

*See the whole thing is diagnosing tuberculosis on the basis of bacteriological diagnosis is something that is done in very small percentage of pediatric tuberculosis. So if that bacteriological diagnosis is not there, it was not being accepted before by BBMP* (Freelancing pediatrician).

### Reinforcement—PPs’ take on incentives for notification

Most of the PPs interviewed across specialties were not aware of monetary incentives for TB notification and did not see the monetary incentive (this is an incentive of Rs.1,000/− [USD 11.43] for private PPs given in 2 instalments—at notification and at treatment completion) as a motivator for greater notification volume. They either felt that the amount of money could be insufficient for a doctor or that the PPs were obliged to the National programme to notify, and incentives were not desirable. A monetary incentive was thought to be appropriate for other categories of PPs, such as pharmacists. Some PPs feared that the incentive option could be misused by deliberate duplication of cases on paper. One PP, however, mentioned that he looked forward to receiving the monetary incentive but had not yet received it.

#### Misuse of incentive provision

*Then people will give false notification. When you give Rs. 500 (USD 5.72), they can do the same thing for 2 samples, duplicate it and get the money. See, people can do anything. Do not you think that is a bad idea?* (Freelancing Internal Medicine specialist).

#### It is the doctor’s duty to notify, no need of incentives

*It is the doctor’s duty to notify. He should not be given any such incentives. If he is given Rs. 500, that is a very wrong idea. It is his duty because he is a doctor. He has to notify, it should be a rule, you must notify the cases to us. An order should be given, not incentives. Then that same Rs. 500 should be passed on to the patient for his proteins, anemia and nutrition and medicines* (General practitioner).

#### Incentive money not sufficient

*If it is paid to upload the data which will take 10 min, then yes, I think so but the question, is it enough, I do not know* (Free lancing pulmonologist).

#### Incentives are not important for doctors but may be motivators for other health care workers

*Probably, not as a doctor but pharmacist or Asha worker, if they are given incentives, they may show more interest in catching more patients and notifying them. But for doctors, it does not make much difference* (Free lancing Pulmonologist).

*I hope it does(result in increased notification volume from PPs) (laughs), to me it does not, but that is very individualistic, so I think especially for pharmacists and some doctors, it may motivate them to remember and notify* (Free lancing pulmonologist).

#### Incentive not reaching doctors

*I have given a case expecting money to come, it is 3 months, and nothing has come. So, they should not lose interest. Things should become more robust… whatever the government has promised, if they can do it, not for me, it may be immaterial for me, but for GPs(general practitioners), where they do private practice, 500 rupees matters definitely* (Free lancing pulmonologist).

### Enabling factors—the facilitators of TB notification

There was a remark by a pediatrician that the NTEP guidelines were eliminating the ambiguity and non-uniformity in the diagnosis of paediatric TB. Statements made by two PPs pointed to expectations they had from the NTEP in the form of reinforcements—first, recognizing PPs for their notification on some professional platforms and second, stringent enforcement of the “compulsory TB notification” provision.

*But that is not a big deal. We do not want it. No need of monetary incentive. Either you recognize our work saying that he is a good TB notifier and you give us some plaque or something. You make a conference or something and award the good notifiers. Not monetary, recognition of the work is sufficient* (Free lancing pulmonologist).

*And fame and appreciation, yes, I think this is a positive step towards knowing about the disease. Coming back to India, first thing should be enforcement. When you club both enforcement and incentives, it is fantastic, it should do* (Freelancing Internal Medicine specialist).

### Barriers to TB notification

The two greatest barriers to notification from PPs were the obligation to maintain confidentiality about their patients and TB patients’ desire for PPs to refrain from sharing their details with anyone due to stigma. This phenomenon was amplified in corporate hospitals where the education level of patients was high. One hospital administrator felt the absence of an NTEP hub in corporate hospitals, unlike medical colleges where these hubs would undertake all TB-related notification work. There was also a lack of knowledge about how TB notification had to be performed even if the PPs wanted to.

#### Obligation to maintain confidentiality hindering notification

*I think the Nikshay app asks for patient identifiers, it is not totally anonymous. Some identifiers are linked and some may not be like to be identified as TB patients* (Pulmonologist, Medical College).

*But unfortunately, what happens is many of the patient did not like it. A couple of them came forward and said without my consent how would you do it and like it happens in many of these hospitals, many of them are totally educated. And in an educated class, they know what are their rights and things, and we had to convince them saying that this is something the government is collecting data, it is nothing that we can hide* (Hospital Administrator).

#### There are no NTEP hubs in corporate hospitals

*We had a Center in the medical college itself whereas in most of the private hospitals, we do not have anything like that* (Hospital Administrator).

#### How to notify?

*It is awareness. They do not know how to notify. See I did not open. Once you get into that, you should get started. I am just postponing. You need someone who has done it* (free lancing pediatrician).

### Suggestions

The PPs offered suggestions to improve TB notifications from the private sector. The suggestions along with the relevant quotes are listed below:

#### List laboratories where testing for TB was being performed and actively seek notification from them

*Why do not you enroll laboratories? There are different specimens, whatever type of tuberculosis diagnosis is done. Why do not we enroll them. Forget about who sent it, for what they sent it…you have diagnosed on histopathology TB, you have diagnosed on microscopy TB, you have diagnosed on Gene Xpert or LPA (line probe assay) or whatever it is…notify all of them* (Pulmonologist, Medical College).

#### Leveraging professional associations to encourage TB notification from its members

*The second thing is if it is done through the association, paediatric association, then it will go a long way. That association will tell, please see that you ensure notification of this. Just like polio and other cases like dengue* (free lancing pediatrician).

#### Ensure that private pharmacies maintain a line-list of patients to whom anti-TB medications were issued and notify all those patients

*Go back and see how many he has reported. If he has prescribed, number of prescriptions is to number of notifications, we do not want to give out that key but… Pharmacies must maintain a line list of patients to whom anti TB medication is dispensed* (Pulmonologist 1, Medical College).

*Prescription should be in duplicate. One the pharmacy will keep with them. Have a line list of patients to whom anti TB medication is dispensed* (Pulmonologist 2, Medical College).

## Discussion

We explored facilitators of and barriers to TB notification and perceptions about monetary incentives for TB notification among PPs in Bengaluru city. The maintenance of confidentiality and TB-related stigma were the most important barriers to TB notification, in addition to a lack of awareness about the process of TB notification. NTEP guidelines were perceived as bringing about uniformity in TB diagnosis, which was a challenge earlier, especially in pediatric TB patients. Recognizing the efforts of PPs in TB notification on professional platforms and implementing compulsory notification in letter and spirit were other perceived facilitators. Actively seeking TB notification from clinical laboratories and pharmacies was proposed as a strategy to increase the volume of TB notifications. Monetary incentives for notifying TB patients were not considered motivators by most of the PPs.

PPs expected some follow-up action on the TB notification data in terms of contact tracing, testing and treatment so that the effort of notification was justified. Feedback about patients referred to the NTEP was also not received by the PPs, which added to the hesitancy to notify them. In a study undertaken in Kerala, India, aimed at identifying barriers and opinions regarding TB notification, the need for feedback about referred patients was an expectation of the majority of PPs. The authors concluded that this feedback could constitute a trust-building strategy between the NTEP and PPs (14). Chadha et al. documented feedback from the NTEP as an enabler contributing to increased TB notification in a southern Indian city (15).

The pediatricians in the private sector expressed difficulty in demonstrating a bacteriological diagnosis of TB among their patients. The NTEP, on the other hand, insisted on a bacteriological diagnosis. This prevented them from notifying the patients based on the clinical diagnosis. Siddaiah et al. reported a lower proportion of pediatric TB patients younger than 15 years in Bengaluru city, India. Similarly, they also reported that sputum-positive patients in general had a significantly greater probability of being notified than sputum-negative TB patients (16). This could point toward obstacles in notifying TB patients diagnosed and putting on anti-TB treatment on clinical grounds. In another study of TB management practices in Mangalore city, southern India, 16% of the pediatricians had put their patients on an anti-TB treatment on a trial basis (empirical treatment), and 22% of the pediatricians had not stressed the use of acid-fast bacilli for the diagnosis of pulmonary TB (17). This also suggested that pediatricians encounter frequent situations where bacteriological diagnosis is not possible.

The PPs in the present study were not aware of monetary incentives for notification of TB. Similar findings have emerged in two mixed-methods studies carried out among private providers in the Maharashtra and Gujarat states of India (7, 8). In another study in Karnataka state, India, Dey et al. also found that PPs who were aware of monetary incentives were also more likely to be aware of Ni-Kshay, the TB notification portal (9). One PP in the present study who was aware of the monetary incentive had not received his monetary incentive even after 3 months after notifying. Sahasrabudhe et al. also found that, after notification, only 17 % of PPs in Pune, Maharashtra state, India, actually received a monetary incentive (7). Interestingly, in their mixed methods study performed in Gujarat, Western India, Rupani et al. encountered a barrier to the utilization of monetary incentives, as some PPs felt that the NTEP could assign additional responsibility to them because they had received the incentive. Most of the PPs in the Gujarat study opined that the amount of monetary incentive was substantial enough to act as a motivator for increasing the notification volume (8). This finding is in contrast to the opinions of the PPs in the present study, who did not find the incentive to be substantial and added that TB control is a responsibility that they had to perform. The authors of the present study find this an unusual finding. However, this opinion of the PPs was not universal, with most specialists (freelancing or in medical colleges) endorsing this view. It is possible that the monetary incentive could still act as an incentive to a few PPs. This view is corroborated by a study undertaken in West Bengal, India wherein 28% of informal providers (providers without a formal medical qualification) expected an incentive for their involvement in NTEP (18). Additionally, increment in the amount of monetary incentive might bring about better motivation. Some PPs in the present study also felt that the monetary incentive option could be misused. In fact, in Pune, Maharashtra State, India, the authors demonstrated situations in which the private laboratory personnel who reported the TB case before the PPs did receive the incentive, although the PPs had sent the patients to the laboratory for diagnostic tests (7). Notably, Philip et al. suggested nonmonetary incentives as a method to boost cohesion and coordination between private providers and the NTEP.

Notification of TB by medical practitioners, clinical laboratories and pharmacies has been made mandatory in India through a gazette notification dated 16^th^ March, 2018 (19). Penal consequences have also been stated in this gazette for those who fail to notify. Some PPs in the present study have observed that the penal provision of this notification is not being implemented on the ground. The authors therefore suggest that implementing the penal provision of notifications in letter and spirit could help increase the volume of TB notifications from the private sector and that notification of TB should be actively pursued from pharmacies and clinical laboratories. The involvement of professional associations such as the Indian Medical Association (IMA) and the Indian Academy of Pediatrics (IAP) to motivate their members to notify them of TB is also proposed. In a review of strategies and achievements of NTEP in engaging PPs and the way forward, Suseela RP and Shannawaz M emphasize that the private sector involved in TB care in India is very wide including the formal and informal private providers, laboratories, pharmacies etc. and specific strategies are needed for engaging them (20). The authors of a mixed methods study aimed at identifying barriers to TB notification in Bengaluru, southern India, have also offered issuing warnings/memos/monetary penalties to defaulters as one of the possible solutions to reinforce the importance of notification (16). In a study carried out in Taiwan, PPs who were interviewed regarding their knowledge, attitudes and practices regarding reportable communicable diseases expressed increased willingness to report if they would receive a reward for reporting or a penalty for not reporting (21).

The four widely experienced barriers to TB notification in the present study were the need to maintain confidentiality about patients, patients’ displeasure of sharing information about their medical condition with the NTEP because of the stigma they experienced, lack of knowledge about the process of TB notification, and, last, the absence of an intermediary who could coordinate the TB notification process at private healthcare facilities other than medical colleges. The stigma barrier was more pronounced in corporate hospitals where the patients were from middle and higher socioeconomic strata. Several studies conducted in the southern Indian states of Kerala (14), Karnataka (9, 15, 16), Tamil Nadu (22) and in an African country (23) have revealed PPs’ barriers pertaining to the maintenance of confidentiality and stigma. Philip et al. also supported the findings of the present study, in which patients from higher socioeconomic strata had heightened concerns about TB-related stigma (14). Similarly, in his commentary on ways and means of utilizing private practitioners for TB care in India, he proposed utilizing the services of the Accredited Social Health Activist (ASHA), a grassroots-level health care functionary in India, as an intermediary between the NTEP and private practitioners (24). A lack of knowledge about the process of notification has been echoed in several studies. Siddaiah et al. reported that providers in a tertiary care private hospital in Bengaluru, southern India, did not know how to notify them or did not perceive TB notification as their responsibility (16). Chadha et al., in their study in Mysore city, southern India, documented that 91% of the PPs were aware of the need to notify; however, only approximately 15% were registered in Ni-Kshay, the TB notification portal, and 23% of PPs looked forward to receiving training in notification through Ni-Kshay, which they considered an enabler (15).

In what could be considered a facilitator of TB notification, in the present study, two PPs preferred to be recognized/honored on a professional platform for their efforts to notify TB patients. This has been corroborated by Ananthakrishnan et al. in their suggestions for way forward as an effective strategy to effectively engage the private sector in TB care (22). A pediatrician who participated in the present study remarked that the NTEP guidelines for the diagnosis and management of TB had simplified their job and had eliminated the uncertainty in the diagnosis and management of pediatric TB. Even though this remark pointed specifically to the availability of a national guideline for pediatric TB, it may be applied as an enabler to the larger context of all forms of tuberculosis. Khan et al., in their attempt to gather lessons learned from initiatives to engage for-profit providers in TB control in South Asia, emphasized that national guidelines are important resources for sharing up-to-date information on managing TB patients and acknowledge the challenge of disseminating these guidelines to all PPs (25).

The theories used as the framework for analysis have provided a comprehensive understanding of beliefs and opinions shaping TB notification from the PPs. The role of self-efficacy in providing a diagnosis has been brought out by the social learning theory and the theory of learned behavior. The fact that there are expectations of the PPs from NTEP is also crucial, the most important expectation being feedback about the cases notified by them. Reinforcement as a construct of social learning theory and its reference to the practice of TB notification through provision of incentive has demonstrated that incentive is not perceived by the PPs as motivators. PPs considered recognition for their performance by NTEP being greater motivators. The precede/proceed model’s constructs of barriers and enabling factors have helped discern the factors which the PPs consider as enablers or deterrents of notification.

The results of the present study have crucial implications for policy and practice in the following domains: feedback to PPs regarding notified TB cases, notifying TB cases based on clinical grounds, firm implementation of compulsory notification provision, enabling PPs to notify through training sessions and recognizing efforts of PPs in Tb notification.

## Conclusion

The utilization of theoretical framework as discussed in methodology has facilitated tangible categorization of perceptions of PPs regarding TB notification into those pertaining to self-efficacy in TB diagnosis and notification, reinforcement of intention to notify, creation of enablers for notification and lastly, expectations of PPs from NTEP. Triangulation of data across the FGDs and IDIs showed consistency of opinions and beliefs across the different specialties and contexts. Further, the dialogue with PPs in the present study has pointed to several possible future directions. Stringent implementation of compulsory notification provisions from all stakeholders can improve credibility of NTEP. Refraining from insistence on bacteriological diagnosis can boost volume of notification from PPs. Setting up a communication channel to provide feedback to PPs about the cases notified by them could help bolster notification from PPs. Since there was a general opinion that incentives were not really motivators for notification, the suggestion of PPs relating to identifying their best practices and honoring them on professional platforms must be considered. Additionally, NTEP may contemplate on augmenting the monetary incentive. Educational interventions for TB patients regarding the necessity to notify TB may curb the stigma attached to TB, thereby making the path to TB notification easier for the PPs. Listing of PPs in each tuberculosis unit under NTEP and scheduling yearly training (online/offline) on TB notification through Nikshay may help overcoming hesitancy to notify. These interactions can also be harnessed to obtain feedback and suggestions from PPs, thereby making them partners in TB control.

## Limitations

The findings of the present study are limited by the fact that they may not be generalizable to contexts other than urban areas of India. Further since the present study uses purposive and snowball sampling, true representation of all PPs might not have been achieved. In addition, since NTEP has been a dynamic programme, certain ground realities might have changed between the time of data collection and publication.

## Data Availability

The raw data supporting the conclusions of this article will be made available by the authors, without undue reservation.
